# Brain-derived synaptic vesicles have an intrinsic ability to sequester tubulin

**DOI:** 10.1186/s12915-025-02464-9

**Published:** 2025-11-14

**Authors:** Tiago Mimoso, Aleksandr Korobeinikov, Alexander Stein, Dragomir Milovanovic, Silvio O. Rizzoli, Sarah Köster, Sofiia Reshetniak

**Affiliations:** 1 Institute for X-Ray Physics, University of Göttingen, Göttingen, Germany; 2 Laboratory of Molecular Neuroscience, German Center for Neurodegenerative Diseases, Berlin and Bonn, Germany; 3 Research Group Membrane Protein Biochemistry, Max Planck Institute for Multidisciplinary Sciences, Göttingen, Germany; 4 Institute of Biochemistry and Einstein Center for Neuroscience, Charité-Universitätsmedizin Berlin, Corporate Member of Freie Universität Berlin, Humboldt-Universität Berlin, and Berlin Institute of Health, Berlin, Germany; 5 Department of Neuro- and Sensory Physiology and Biostructural Imaging of Neurodegeneration (BIN) Center, University Medical Center Göttingen, Göttingen, Germany; 6 Cluster of Excellence “Multiscale Bioimaging: From Molecular Machines to Networks of Excitable Cells” (MBExC), University of Göttingen, Göttingen, Germany

**Keywords:** Synapse, Synaptic vesicles, Synaptic vesicle cluster, Tubulin, Microtubules, Cytoskeleton

## Abstract

**Background:**

The presence and function of microtubules within the synaptic bouton has long been under investigation. In recent years, evidence has accumulated that connects the synaptic vesicle cluster to the local dynamics of microtubule ends. Nonetheless, one question remains open, namely whether the vesicles influence the availability of tubulin within the synaptic compartment.

**Results:**

An analysis of previously published live imaging experiments indicates that tubulin is strongly enriched in the synaptic vesicle cluster. To analyze the vesicle-tubulin interaction directly, we isolated vesicles from the mouse brain and imaged them together with fluorescent tubulin in vitro. We found that soluble tubulin is collected by synaptic vesicles in physiological buffers, resulting in the formation of tubulin-rich regions (TRRs) on the respective vesicle clusters.

**Conclusions:**

We conclude that the synaptic vesicle cluster is indeed able to recruit soluble tubulin.

**Supplementary Information:**

The online version contains supplementary material available at 10.1186/s12915-025-02464-9.

## Background

Synaptic transmission relies on the fusion of neurotransmitter-loaded synaptic vesicles (SVs) with the plasma membrane of the synaptic bouton to deliver their contents into the synaptic cleft and to thus activate postsynaptic receptors. The presence of SVs in the synaptic bouton is ensured by cytoskeleton-dependent transport of SV precursors, which are continually brought from the cell body and are assembled into vesicles in the synapse [1, 2]. Their interaction with microtubules and actin cytoskeleton continues throughout their lifetimes, as vesicles are continually transported between synapses [3–6] or even between synapses and the cell body [7]. Recent works demonstrate that while the majority of SV employ actin cytoskeleton, a population of mature SVs rely on microtubules for their transport [6], proposedly as they are targeted for trafficking back to the soma for degradation [8].

While the transport-related interaction of SVs and microtubules is therefore well-established, with the presence of microtubules heavily influencing either the delivery of SV precursors to synapses or SV transport to the cell body, other effects of microtubules on SV dynamics are less clear. A detailed analysis of neurotransmission at a large central synapse, the calyx of Held, led to the observation that microtubules enter deep into the synapse, where they colocalize with a large number of resident (not transported) SVs [9], which fits well with many previous analyses of synapses by electron microscopy (e.g., [10, 11]). Moreover, microtubule depolymerization slowed down the recovery of the synapses from short-term depression [9], suggesting that such microtubules exhibit a functional interaction with SV clusters.

In principle, functional interactions between microtubules and SVs could take many other additional forms. The protein tau, well known from the context of diseases termed tauopathies (including Alzheimer’s disease), is mainly considered to be a stabilizer of microtubules [12]. Tau is recruited to synapses, at least in pathological conditions, by binding to the SV membrane proteins synaptogyrin-3 [13], where it seems to alter actin and microtubule assembly, by causing a local de novo polymerization, with deleterious effects on synaptic function [14, 15]. Notably, the effects of tau mutations on synaptic transmission differ between animal models [16], and tubulin binding adds another complicating factor to be considered in this area.

Other SV proteins may interact with tubulin, including highly abundant molecules as the calcium sensor synaptotagmin I, whose calcium-binding domains bind directly to the C-terminal region of beta-tubulin, and can cause tubulin polymerization in vitro [17]. Two soluble synaptic proteins, which associate closely to SVs, synapsin I and alpha-synuclein, also bind microtubules, with the first being able to bring microtubules together, in bundles [18], while the latter appears to increase the growth rate of microtubules, as well as the frequency of rapid shrinkage period (also termed microtubule catastrophe events [19]).

Overall, these observations suggest that SVs may interact with tubulin, causing the retention of soluble tubulin within the presynaptic space, and thereby modulating the local microtubule dynamics. This is further suggested by an analysis of the distribution of tubulin in the synapse, and of its amounts, in relation to the SV density [20]. Tubulin density enriches non-linearly with the SV density, suggesting that this protein indeed preferentially locates to SV clusters. However, other scenarios could also be imagined, under different synapse function conditions, with microtubule polymerization creating tubulin sinks away from the synapse, where limited levels of soluble tubulin remain.

To investigate whether SVs can bind soluble tubulin, we first considered the distribution of soluble tubulin in pre-synapses, by reanalyzing our data from [21] and then experimentally investigating the interaction of SVs, isolated from rodent brains, with purified tubulin, by different imaging analyses. We found that the SVs were able to collect tubulin, confirming the previous fluorescence recovery after photobleaching (FRAP)-derived observations, and suggesting that SV-induced tubulin accumulation in synapses may be an important parameter for both synaptic and microtubule dynamics.

## Results

**Fig. 1:**
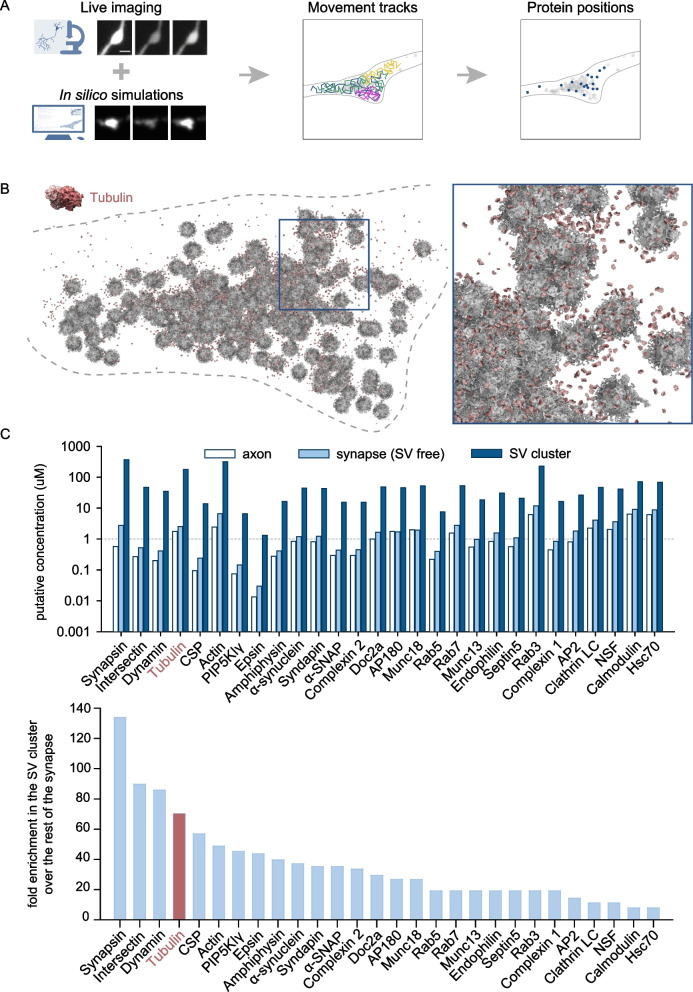
Tubulin distribution in the synaptic bouton.** A** Schematic explanation of the experimental workflow of [21]. Live imaging was combined with in silico simulations to generate individual protein movement tracks, from which the positions of molecules could be extracted. **B** Visualization of soluble tubulin dimers in a synaptic bouton, based on results from [21]. Dashed line indicates synapse borders. Inset: a zoom-in into the synaptic vesicle cluster, demonstrating a higher local density of tubulin molecules. **C** Quantification of the concentration of various soluble proteins in axons, SV-free synaptic space, and the SV cluster (top) and of the molar enrichment of respective proteins in the SV cluster, compared to the synaptic space outside of the cluster. Tubulin is among the most enriched proteins

Our own observations of soluble, green fluorescent protein (GFP)-tagged tubulin [21], expressed in cultured hippocampal neurons, suggest that abundant levels of soluble tubulin are present in the synapses. Using the data from the mentioned study, we performed further analysis of the distribution of soluble tubulin in the synaptic bouton, which demonstrated higher local density of tubulin molecules in the vicinity of the SVs (Fig. 1A, B). Indeed, calculations of the molar enrichment of various proteins in the synapse suggests that tubulin enriches strongly in the SV cluster, being the 4th-most enriched molecule in this environment (Fig. 1C). This is an unexpected observation, since the magnitude of the enrichment of tubulin in the SV cluster is substantially higher than for *bona fide* SV-binding molecules, as Rab3, or for vesicle cluster components, as alpha-synuclein (Fig. 1C).

**Fig. 2:**
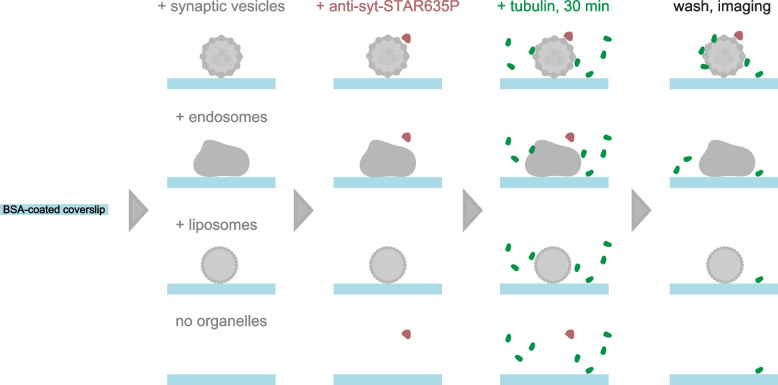
Scheme of the experimental workflow. Synaptic vesicles are allowed to adhere onto BSA-coated glass coverslips, and are immunostained with fluorescently labeled anti-synaptotagmin nanobodies. A buffer containing native and fluorescently labeled tubulin is then added, and is incubated for 30 min at 37 °C. All unbound proteins are then washed off, and the samples are analyzed by fluorescence imaging. The same procedure is performed in the presence of fluorescently labeled endosomes, liposomes, or in the absence of any organelles, as controls

This computational analysis suggests that SVs might accumulate soluble tubulin on their surface. To directly observe this possible interaction of soluble tubulin with SVs, we relied on a simple setup, as illustrated in Fig. 2. We immobilized purified brain-derived SVs (from the brains of adult mice) on glass coverslips and incubated them with a solution of purified tubulin (5% fluorescently labeled). We then analyzed the distribution of tubulin, relative to the SVs, by fluorescence microscopy.

**Fig. 3:**
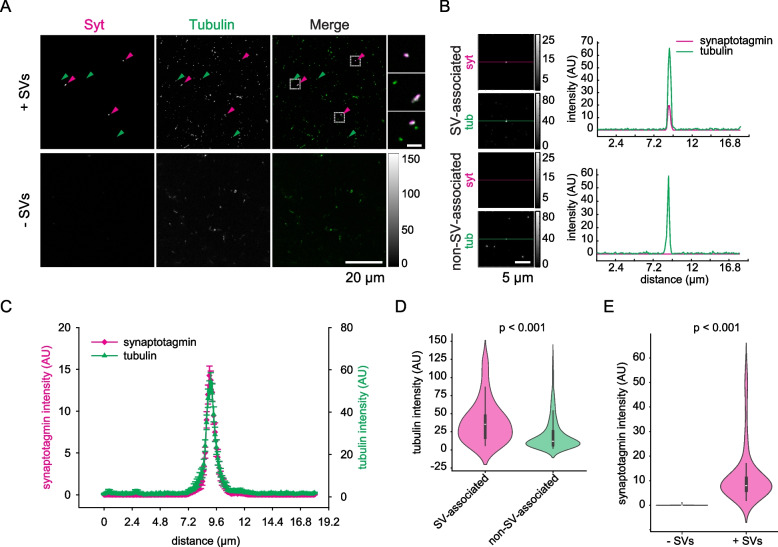
Tubulin accumulates in SV-enriched areas.** A** Confocal view of tubulin distribution in the presence and in the absence of SVs. Arrowheads indicate SV-associated (magenta) and non-SV-associated TRRs (green). Zoom-ins into the squared regions are shown on the right. Inset scale bar: 2 μm.** B** Left: exemplary images with automatically detected TRRs in the center and corresponding synaptotagmin signal. Colored lines indicate the position where line scans are taken 75 pixels in both directions from the center of the identified spot. TRR are classified into SV-associated (top) or non-SV-associated (bottom) based on the intensity of the synaptotagmin signal in the region masked by tubulin channel. Right: line scans from the regions showed on the left. **C** Average line scans indicating tubulin signals overlapping with SVs, as detected in confocal imaging. **D** Comparison of tubulin intensity in TRRs associated or not associated with SVs. Data from 5 independent experiments, 55 SV-associated TRRs and 540 non-SV-associated TRRs were quantified. Indicated significance bracket: Kruskal–Wallis test, *p* = 0.0008. **E** Synaptotagmin intensity in samples with or without SVs. Data from 3 independent experiments, 89 TRRs from samples containing SVs and from samples not containing SVs were quantified. Wilcoxon rank sum test, *p* = 9.8111 × 10.^−34^

We found that soluble tubulin tends to accumulate in distinct loci, which we term tubulin-rich regions (TRRs). These TTRs often, but not always, colocalize with the clusters of SVs, and are also formed in the absence of the SVs (Fig. 3A, Additional file 1: Fig. S1A,), implying that two populations of TRRs form, SV-associated and SV-non-associated ones. Average line scans through automatically selected synaptotagmin-positive loci indicate a clear overlap between the SVs and SV-associated TRRs (Fig. 3B, C, Additional file 1: Fig. S1B).

SV-associated TRRs had a higher intensity than TRRs forming in the same samples, but in SV-free areas (Fig. 3D, Additional file 1: Fig. S1C). The intensity of the latter was also lower than that of TRRs forming in the absence of SVs (in samples containing only tubulin, but no other molecules), as indicated in Additional file 1: Fig. S1C. This observation suggests that SVs sequester tubulin, reducing the availability of these molecules for the formation of TRRs in other regions. To validate the SV interaction to the TRRs, we quantified the intensity of anti-synaptotagmin staining in the presence and in the absence of SVs. The results suggest that the colocalization observed between TRRs and SVs is not affected by autofluorescence or other artefacts (Fig. 3E, Additional file 1: Fig. S1D).

Notably, in both widefield and confocal images, we only sporadically observed short microtubules, suggesting that the interaction of SVs and tubulin may not automatically translate to microtubule polymerization, in spite of the presence of paclitaxel, which should stabilize newly formed microtubules.

**Fig. 4:**
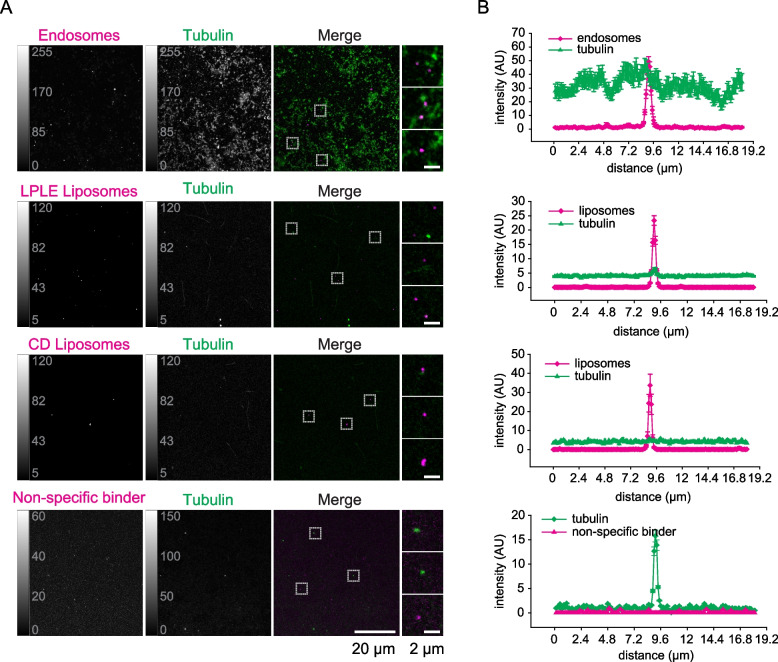
Tubulin does not preferentially accumulate on endosomes, artificial vesicles, or at sites of non-specific protein adhesion.** A** Confocal views of tubulin distribution in samples where fluorescently labeled endosomes, two types of liposomes (LPLE, liver polar lipid extract; CD, chemically defined composition, 49.8:25:10:15:0.2 POPC:POPE:POPS:cholesterol:Texas Red DHPE), or non-specific binder (anti-rabbit antibody, used with no rabbit antibodies in the sample) were attached to the coverslips instead of synaptic vesicles. **B** Averaged line scans from the corresponding samples. Data from 3 independent experiments, 30 endosome-positive regions, 178 LPLE-liposome-positive regions, 25 CD-liposome-positive regions, 86 tubulin-positive regions for the non-specific stainings

In principle, these observations could be explained by the non-specific interaction of tubulin with any membranes provided, or by the binding of both tubulin and SVs to adherent patches on the coverslips. To test the first of these hypotheses, we repeated the experiments in the presence of fluorescently labeled endosomes, isolated from HEK293 cells, or artificial liposomes of various compositions instead of SVs. As shown in Fig. 4A, tubulin demonstrated seemingly random distribution, with no clear preferential localization close to endosomes or liposomes. The intensity correlation analysis indicated virtually no colocalization with liposomes of either chemically defined (CD) composition or derived from a liver polar lipid extract (LPLE), and moderate, but considerably less pronounced compared to the SVs, colocalization with cell-derived endosomes (Fig. 4B, Additional file 1: Fig. S2). The significantly increased amount of tubulin retained in the endosome-containing sample can be explained by its binding to other cellular components present in the endosomal preparation, which are, however, not fluorescently labeled.

To test the second hypothesis, of the possible coincidental binding of both tubulin and SVs to adherent patches on the coverslips, we incubated the coverslips with tubulin and a fluorescently labeled antibody, in the absence of an antigen, to reveal possible locations of non-specific binding on the coverslip surface. An analysis of the relative distribution of TRRs and the non-specific binder revealed effectively zero overlap between these signals (Fig. 4B, Additional file 1: Fig. S2).

Apart from the non-specific adherence of tubulin to the coverslips, the observed signal accumulations might be mediated by fluorophore interactions. To test this, we repeated the experiments using a lower tubulin labeling ratio, while preserving the same total tubulin concentration. This resulted in preserved colocalization, but decreased tubulin signal intensity (Additional file 1: Fig. S3), indicating that non-labeled tubulin outcompetes the labeled molecules, therefore confirming the role of tubulin, not the fluorophore, in establishing this interaction. Lastly, we tested whether visualizing SVs by targeting another SV protein will affect the relative distribution of SV and tubulin signal. We repeated the experiments, immunostaining for synaptophysin, which resulted in tubulin accumulation around synaptophysin-positive regions (Additional file 1: Fig. S3). Taken together, these experiments indicate that the colocalization of SVs and tubulin is caused by a specific interaction between them.

## Discussion

The presence of tubulin cytoskeleton in the presynapse has been well established, starting with early electron microscopy observations of synaptic boutons [22]. Microtubules can serve as “tracks” for antero- and retrograde transport of synaptic components, and have been observed to enter the synaptic vesicle cluster and approach the active zone [9, 11, 23]. Notably, presynapses are particularly rich in dynamic microtubule plus-ends, and act as hotspots for activity-induced microtubule nucleation [24, 25].

This presence of highly dynamic microtubules, associated with the synaptic vesicle cluster, suggests that the synaptic vesicle cluster might also contain significant amount of microtubule building blocks, i.e., soluble tubulin, which would support local microtubule polymerization. Indeed, our data places soluble tubulin as the fourth most-enriched protein in the synaptic vesicle cluster (Fig. 1C), surpassed only by the SV-binding protein synapsin, the scaffold intersectin, and the endocytosis cofactor dynamin. The abundance of synapsin, intersectin, and dynamin in the synaptic vesicle cluster is expected, as these molecules are highly abundant in the synapse, [20], and have been implicated in vesicle interactions in the past [26].

Moreover, these molecules, along with SVs, are thought to form the synaptic vesicle cluster, through a process of liquid–liquid phase separation (LLPS; [26, 27]). LLPS-formed organelles are known to maintain their composition by locally increasing the concentration of the participating proteins, while excluding others. Many of the proteins enriched in the synaptic vesicle cluster either have been shown to participate in the LLPS, or have structural motifs enabling them to do so. Tubulin, however, is an exception, since it has not been shown to participate in LLPS processes in the synapse, implying that the mechanisms for its strong enrichment in the synaptic vesicle cluster remain unclear.

Here we demonstrated that isolated SVs are able to collect soluble tubulin. This observation fits well with the known presence of dynamic microtubules and the high enrichment of soluble tubulin within the SV clusters. Tubulin’s recruitment to SVs may be due to direct interactions with SV membrane components (as synaptotagmin), or to interactions with synapsin molecules, as discussed in the Introduction. As these molecules are found to levels of ~ 8 copies per purified SV [28], they would certainly be able to recruit tubulin.

**Fig. 5:**
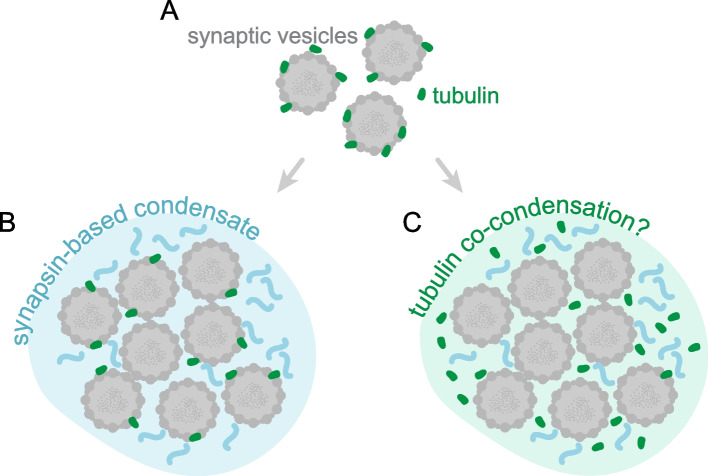
Possible organization of tubulin in the synaptic vesicle cluster.** A** Our data demonstrates that soluble tubulin binds free synaptic vesicles. **B** According to the currently accepted model of the synaptic vesicle cluster formation, SVs, together with SV-binding protein synapsin, form distinct condensates via LLPS. As tubulin binds to the SVs, it is presumably present in the condensates formed by them. **C** It is also conceivable that tubulin might be an active participant in the phase transitions [30–32], therefore in this panel it is depicted as a component of the condensate, not strongly bound to synaptic vesicles directly, but rather switching between a bound and an unbound state, typical for LLPS formation

Nonetheless, it remains unclear whether tubulin participates in synaptic LLPS (Fig. 5). Neither FRAP experiments, nor common LLPS-manipulating drugs, like 1,6-hexanediol, can provide a clear answer. This is because the behavior of the molecules could be explained both by *bona fide* LLPS arrangements and by binding and unbinding from low-mobility objects, such as SVs. In living cells, this ambiguity is difficult to resolve [29]. Moreover, tubulin may exhibit complex dynamics in synapses, separating into freely soluble, SV-bound and polymerized pools of molecules. This behavior will not be easily tracked in either FRAP or 1,6-hexanediol treatments, leaving this topic in need of new experimental approaches, to provide a thorough answer.

It is also not clear which interaction specifically drives tubulin accumulation in the SV-rich areas: as mentioned above, tubulin binding can be supported by both interactions with synaptic vesicle proteins (e.g., synaptotagmin) and with SV-binding proteins, such as synapsin or alpha-synuclein. Depending on which interaction prevails in vivo, it might lead to different functional roles in synaptic physiology. For example, accumulation of tubulin via weak, but frequent, interactions with soluble proteins would lead to its local increase in concentration, possibly promoting microtubule nucleation within the synaptic vesicle cluster. Alternatively, strong binding to SVs themselves might limit the availability of tubulin monomers, effectively creating a tubulin sink, thereby inhibiting microtubule growth. This latter hypothesis is in line with our observation that the SV-bound tubulin rarely generates microtubules, even in the presence of paclitaxel, which should promote microtubule formation.

Overall, it is quite likely that several of the proposed processes occur simultaneously and the balance between them is regulated, to accommodate changes in activity levels.

### Limitations of this study

The experiments reported here were performed in a simplified in vitro system, with typical limitations of such approaches. First, they do not consider contributions of other cellular components that might modulate SV-tubulin interactions. Tubulin concentration and SV density do not recapitulate such of intrasynaptic ones, which might also have an effect on the dynamics of their interaction. Additionally, the mixed sourcing of the SVs, endosomes, and tubulin might affect the interactions due to differences in, e.g., post-translational modifications between model systems. Lastly, our SV purification protocol does not discriminate between neurotransmitter carrying vesicle type, meaning that it is impossible to state whether tubulin-binding ability of SVs is limited to synapses of a specific type, based on the results presented here. The current work represents a proof-of-concept study and further investigations of SV-tubulin interactions focused on their analysis in native conditions of a synapse are warranted.

## Conclusions

Our results indicate that isolated synaptic vesicles are able to recruit soluble tubulin, providing a mechanism for tubulin enrichment in the synaptic vesicle cluster.

## Methods

### Analysis of soluble tubulin distribution in synapses

Experimental data was retrieved from [21]. In short, protein mobility data was collected using FRAP on synapses of cultured neurons, while electron microscopy provided detailed synaptic morphology reconstruction. A 3D synapse model was constructed, where particle movement was simulated by modeling particle trajectories, incorporating parameters as diffusion speed and SV binding, to reproduce the experimental fluorescence recovery recordings. The generated individual protein trajectories were then combined with quantitative data on protein abundancies from [20], to generate detailed map of protein distribution in synapses. For more details on data acquisition and modeling, refer to the original work [21].

Using the previously generated data on protein abundancies, positions, and synaptic geometry, we then calculated the local concentrations of various soluble proteins in axons, SV-free synaptic space, and within the SV cluster, which we define as the space occupied by the SVs and their immediate surrounding of twice their size. Using the obtained number, we then calculated the enrichment of respective proteins in the SV cluster, as reported in Fig. 1.

### Synaptic vesicle purification

Murine SVs were purified using the modified protocol from [33–36]. In brief, adult C57BL/6 J mice (1:2 male:female ratio) were sacrificed by cervical dislocation and their whole brains including cerebellum extracted and homogenized in the homogenization buffer: 320 mM Sucrose, 5 mM HEPES–NaOH, pH 7.3, supplemented with 0.2 mM PMSF and 1 mg/mL Pepstatin A. Cellular debris was removed by centrifugation at 900 g, while supernatant was further centrifuged at 12,000 g. The white synaptosome-containing pellet was carefully washed with Homogenization buffer and centrifuged at 14,500 g. The obtained pellet was subjected to a hypo-osmotic lysis in the lysis buffer: 5 mM HEPES–KOH, 0.2 mM PMSF, 1 mg/mL Pepstatin A, pH 7.3. Lysate was centrifuged at 32,500 g to pellet the contaminants and SV-containing supernatant was further ultracentrifuged at 247,000 g. Pellet was resuspended in 40 mM sucrose and fractionated on a continuous sucrose density gradient (50–800 mM sucrose) by ultracentrifugation in SW 40 Ti rotor (Beckman) at 29,500 RPM for 3 h. All sucrose solutions were buffered with 5 mM HEPES–KOH, pH 7.3. SV-containing fraction was collected from the gradient, pelleted by centrifugation at 247,000 g, resuspended in the final reaction buffer: 150 mM NaCl, 25 mM Tris–HCl, 0.5 mM TCEP, pH 7.4 after which it was snap-frozen in liquid nitrogen and stored at − 80 °C until use.

The quality control of SVs was validated by Western Blot. Fractions were taken from SV prep brain homogenate, supernatant and pellet after lysate centrifugation and final SVs. All fractions were resolved in 12% Tris–Glycine SDS-PAGE gel and transferred onto Amersham Hybond LFP membrane (0.2 µm pore size, Cytiva). Wet transfer was done overnight at 10 V and 4 °C. Membrane was washed in TBS and blocked in 5% milk or 3% BSA in TBS (depending on the primary antibody combination) for 1 h at room temperature. Incubation with primary antibodies was done overnight at 4 °C with gentle shaking. Antibodies used: VAMP2 (SySy 104,211 1:8000 in 3% BSA/TBS), Synaptophysin 1 (SySy 101,011 1:1000 in 3% BSA/TBS), SDHA (EMD Millipore MABN630 1:1000 in 5% milk/TBS), PSD95 (SySy 124,011 1:1500 in 5% milk/TBS). Staining with HRP-conjugated secondary antibodies (anti-mouse Sigma A4416 1:5000 in 5% milk/TBS or 3% BSA/TBS depending on primary antibody combination) was performed for 1 h at room temperature. Final blot was developed using Amersham ECL Prime Western Blotting Detection Reagents (Cytiva) and imaged on Fusion FX device (Vilber).

### Endosome purification

Endosomes of HEK293 cells were fluorescently labeled by uptake of Alexa Fluor 594-Dextran and isolated as described previously [37].

### Liposome preparation

The following lipids were purchased from Avanti Polar Lipids: POPC (1-palmitoyl-2-oleoyl-sn-glycero-3-phosphocholine), POPE (1-palmitoyl-2-oleoyl-sn-glycero-3-phosphoethanolamine), POPS (1-palmitoyl-2-oleoyl-sn-glycero-3-phospho -L-serine), bovine liver polar lipid extract, and cholesterol. Texas Red DHPE (Texas Red 1,2-Dihexadecanoyl-sn-glycero-3-phosphoethanolamine) was purchased from Thermo Fisher. Large unilamellar liposomes were prepared by reverse-phase evaporation as described [38]. For the chemically defined (CD) mix, lipids were mixed in chloroform at a molar ratio of 49.8:25:10:15:0.2 (POPC:POPE:POPS:cholesterol:Texas Red DHPE). Liver polar lipid extract (LPLE) was supplemented with an approximately matching amount of Texas Red DHPE. Chloroform was subsequently removed using a rotary evaporator by lowering the pressure step-wise to 20 mbar. The lipid film was then dissolved in diethyl ether to a final concentration of 20 mM. Three hundred microliters of buffer (20 mM HEPES/KOH pH 7.4, 150 mM potassium chloride) was added, and the sample was sonicated for 1 min on ice (Branson Sonifier 450, 100% duty cycle, microtip limit 1). Afterwards the ether was removed at 500 mbar. After 10 min, additional 700 µL of buffer was added and the pressure was gradually decreased to 100 mbar until diethyl ether was completely removed. The resulting lipid suspension was extruded through a polycarbonate filter (11 × through a 0.4-µM filter, 21 × through a 0.1-µM filter) using the mini extruder kit (Avanti Polar Lipids).

### SV*-*tubulin in vitro interaction assay

Circular 18 mm No. 1.5 glass coverslips were coated with BSA by incubation in 5% BSA in PBS overnight. One femtomole of SVs per coverslip was added and centrifuged for 1 h at 4000 g to force SV adhesion to the coverslips. Supernatant with unbound SVs was removed and remaining SVs were stained with 25 nM FluoTag®-X2 anti-Synaptotagmin 1 coupled to Abberior Star 635P (NanoTag Biotechnologies, Göttingen, Germany) for 30 min in a humidifying chamber at room temperature. The coverslips were then washed with PBS containing 2.5% BSA, followed by a wash with General Tubulin Buffer (80 mM PIPES pH 6.9, 2 mM MgCl_2_, 0.5 mM EGTA) (Cytoskeleton, Inc, Denver, USA) supplemented with 10% glycerol and 1 mM GTP. The coverslips were then incubated with 5 mg/ml tubulin solution, consisting of 95% unlabeled porcine brain tubulin (Cytoskeleton, Inc, Denver, USA) and 5% fluorescently labeled (HiLyte Fluor™ 488) tubulin (Cytoskeleton, Inc, Denver, USA) in General Tubulin Buffer supplemented with 10% glycerol and 1 mM GTP for 30 min at 37 °C. Following the incubation, unbound tubulin was washed off using stabilization buffer (General Tubulin Buffer supplemented with 30% glycerol, 1 mM GTP, and 5 μM paclitaxel) and any microtubules formed on the SV were stabilized by incubation in stabilization buffer for 5 min at 37 °C. The samples were then fixed with 4% PFA in PBS for 20 min, quenched with 100 mM NH_4_Cl in PBS for 20 min and mounted in Mowiol for further microscopic evaluation.

In parallel, control experiments were performed following the same protocol, but in the absence of the SVs, with fluorescently labeled endosomes instead of the SVs, or using non-specifically binding antibodies.

### Microscopy and image processing

Widefield images were obtained using an inverted Nikon Ti-E epifluorescence microscope (Nikon Corporation, Chiyoda, Tokyo, Japan) equipped with a 60 × 1.4 NA oil-immersion Plan Apochromat objective (Nikon Corporation, Chiyoda, Tokyo, Japan), an HBO‐100W Lamp and an IXON X3897 Andor (Belfast, Northern Ireland, UK) camera.

Confocal images were obtained using a TCS SP5 microscope (Leica, Wetzlar, Germany) equipped with a HCX Plan Apochromat 100 × 1.4 NA oil-immersion objective. The 488 nm line of an Argon laser was used for excitation of HiLyte Fluor™ 488, while Alexa Fluor 594 and Abberior Star 635P were excited using HeNe 594 nm and HeNe 633 nm lasers, respectively. Images were collected at 512 × 512 pixel resolution and physical size of 62 × 62 µm. Acquisition settings were kept identical across experiments.

Exemplary images were processed in ImageJ 1.54f (NIH, USA). For quantification, custom-written MATLAB (The MathWorks Inc, Natick, MA, USA) routines were employed. The images were subjected to an empirically defined, automated thresholding procedure, to isolate tubulin-positive regions. The center-of-mass of the respective regions was determined automatically, in the tubulin channel, and line scans were performed automatically, through the pixel corresponding to the center of mass. The peak intensity was measured for tubulin and for the other channels, from the respective line scans. The intensities observed were used to classify the TRRs into SV-associated or non-associated classes, according to the synaptotagmin signals, and to measure the non-specific signals associated to the tubulin spots.

## Supplementary Information

Additional file 1: Fig. S1. Tubulin accumulates in SV-enriched areas. Fig. S2. Pearson correlation coefficients of tubulin and various organelles’ intensity. Fig. S3. Comparison of average line profiles of tubulin and SV marker intensities of TRRs detected in samples using different labeling approaches

## Data Availability

The experimental data that support the findings of this study are available in Figshare with the identifier 10.6084/m9.figshare.30285094. The data generated from the analysis of results in reference [21] are presented within this article. The data generated in reference [21] can be obtained from the respective corresponding author.

## Abbreviations

CD: Chemically defined
DHPE: 1,2-Dihexadecanoyl-sn-glycero-3-phosphoethanolamine
FRAP: Fluorescence recovery after photobleaching
GFP: Green fluorescent protein
LLPS: Liquid-liquid phase separation
LPLE: Liver polar lipid extract
POPC: 1-Palmitoyl-2-oleoyl-sn-glycero-3-phosphocholine
POPE: 1-Palmitoyl-2-oleoyl-sn-glycero-3-phosphoethanolamine
POPS: 1-Palmitoyl-2-oleoyl-sn-glycero-3-phospho-L-serine
SV: Synaptic vesicle
TRR: Tubulin-rich region

## References

[1] Chenouard N, Xuan F, Tsien RW. Synaptic vesicle traffic is supported by transient actin filaments and regulated by PKA and NO. Nat Commun. 2020;11:5318. 33087709 10.1038/s41467-020-19120-1 PMC7578807

[2] Petzoldt AG. Presynaptic precursor vesicles—cargo, biogenesis, and kinesin-based transport across species. Cells. 2023;12:2248. 37759474 10.3390/cells12182248 PMC10527734

[3] Darcy KJ, Staras K, Collinson LM, Goda Y. Constitutive sharing of recycling synaptic vesicles between presynaptic boutons. Nat Neurosci. 2006;9:315–21. 16462738 10.1038/nn1640

[4] Staras K, Branco T, Burden JJ, Pozo K, Darcy K, Marra V, et al. A vesicle superpool spans multiple presynaptic terminals in hippocampal neurons. Neuron. 2010;66:37–44. 20399727 10.1016/j.neuron.2010.03.020 PMC2908741

[5] Gramlich MW, Klyachko VA. Actin/Myosin-V- and activity-dependent inter-synaptic vesicle exchange in central neurons. Cell Rep. 2017;18:2096–104. 28249156 10.1016/j.celrep.2017.02.010

[6] Parkes M, Landers NL, Gramlich MW. Recently recycled synaptic vesicles use multi-cytoskeletal transport and differential presynaptic capture probability to establish a retrograde net flux during ISVE in central neurons. Front Cell Dev Biol. 2023;11:1286915. 38020880 10.3389/fcell.2023.1286915 PMC10657820

[7] Wong MY, Zhou C, Shakiryanova D, Lloyd TE, Deitcher DL, Levitan ES. Neuropeptide delivery to synapses by long-range vesicle circulation and sporadic capture. Cell. 2012;148:1029–38. 22385966 10.1016/j.cell.2011.12.036 PMC3294265

[8] Ivanova D, Cousin MA. Synaptic vesicle recycling and the endolysosomal system: a reappraisal of form and function. Front Synaptic Neurosci. 2022;14:826098. 35280702 10.3389/fnsyn.2022.826098 PMC8916035

[9] Babu LPA, Wang H-Y, Eguchi K, Guillaud L, Takahashi T. Microtubule and actin differentially regulate synaptic vesicle cycling to maintain high-frequency neurotransmission. J Neurosci. 2020;40:131–42. 31767677 10.1523/JNEUROSCI.1571-19.2019 PMC6939482

[10] Gray EG, Westrum LE, Burgoyne RD, Barron J. Synaptic organisation and neuron microtubule distribution. Cell Tissue Res. 1982;226:579–88. 7139692 10.1007/BF00214786

[11] Gray EG, Katz B. Presynaptic microtubules and their association with synaptic vesicles. Proc Biol Sci. 1997;190:369–72. 240166

[12] Barbier P, Zejneli O, Martinho M, Lasorsa A, Belle V, Smet-Nocca C, et al. Role of tau as a microtubule-associated protein: structural and functional aspects. Front Aging Neurosci. 2019;11:00204. 10.3389/fnagi.2019.00204 PMC6692637 31447664

[13] McInnes J, Wierda K, Snellinx A, Bounti L, Wang Y-C, Stancu I-C, et al. Synaptogyrin-3 mediates presynaptic dysfunction induced by tau. Neuron. 2018;97:823-835.e8. 29398363 10.1016/j.neuron.2018.01.022

[14] Zhou L, McInnes J, Wierda K, Holt M, Herrmann AG, Jackson RJ, et al. Tau association with synaptic vesicles causes presynaptic dysfunction. Nat Commun. 2017;8:15295. 28492240 10.1038/ncomms15295 PMC5437271

[15] Hori T, Eguchi K, Wang H-Y, Miyasaka T, Guillaud L, Taoufiq Z, et al. Microtubule assembly by tau impairs endocytosis and neurotransmission via dynamin sequestration in Alzheimer’s disease synapse model. Elife. 2022;11:e73542. 35471147 10.7554/eLife.73542 PMC9071263

[16] Taipala E, Pfitzer JC, Hellums M, Reed MN, Gramlich MW. RTg(TauP301L)4510 mice exhibit increased VGlut1 in hippocampal presynaptic glutamatergic vesicles and increased extracellular glutamate release. Front Synaptic Neurosci. 2022;14:925546. 35989711 10.3389/fnsyn.2022.925546 PMC9383415

[17] Honda A, Yamada M, Saisu H, Takahashi H, Mori KJ, Abe T. Direct, Ca2+-dependent interaction between tubulin and synaptotagmin I. J Biol Chem. 2002;277:20234–42. 11925429 10.1074/jbc.M112080200

[18] Baines AJ, Bennett V. Synapsin I is a microtubule-bundling protein. Nature. 1986;319:145–7. 2417124 10.1038/319145a0

[19] Cartelli D, Aliverti A, Barbiroli A, Santambrogio C, Ragg EM, Casagrande FVM, et al. Α-synuclein is a novel microtubule dynamase. Sci Rep. 2016;6:33289. 27628239 10.1038/srep33289 PMC5024109

[20] Wilhelm BG, Mandad S, Truckenbrodt S, Kröhnert K, Schäfer C, Rammner B, et al. Composition of isolated synaptic boutons reveals the amounts of vesicle trafficking proteins. Science. 2014;344:1023–8. 24876496 10.1126/science.1252884

[21] Reshetniak S, Ußling J-E, Perego E, Rammner B, Schikorski T, Fornasiero EF, et al. A comparative analysis of the mobility of 45 proteins in the synaptic bouton. EMBO J. 2020;39:e104596. 32627850 10.15252/embj.2020104596 PMC7429486

[22] Schmitt FO. Fibrous proteins–neuronal organelles. Proc Natl Acad Sci U S A. 1968;60:1092–101. 5244734 10.1073/pnas.60.4.1092 PMC224885

[23] Lepicard S, Franco B, de Bock F, Parmentier M-L. A presynaptic role of microtubule-associated protein 1/Futsch in *Drosophila*: regulation of active zone number and neurotransmitter release. J Neurosci. 2014;34:6759–71. 24828631 10.1523/JNEUROSCI.4282-13.2014 PMC6608111

[24] Guedes-Dias P, Nirschl JJ, Abreu N, Tokito MK, Janke C, Magiera MM, et al. Kinesin-3 responds to local microtubule dynamics to target synaptic cargo delivery to the presynapse. Curr Biol. 2019;29:268-282.e8. 30612907 10.1016/j.cub.2018.11.065 PMC6342647

[25] Qu X, Kumar A, Blockus H, Waites C, Bartolini F. Activity-dependent nucleation of dynamic microtubules at presynaptic boutons controls neurotransmission. Curr Biol. 2019;29:4231-4240.e5. 31813605 10.1016/j.cub.2019.10.049 PMC6917861

[26] Reshetniak S, Bogaciu CA, Bonn S, Brose N, Cooper BH, D’Este E, et al. The synaptic vesicle cluster as a controller of pre- and postsynaptic structure and function. J Physiol. 2024; 10.1113/JP286400 PMC12559990 39367860

[27] Hoffmann C, Rentsch J, Tsunoyama TA, Chhabra A, Aguilar Perez G, Chowdhury R, Trnka F, Korobeinikov AA, Shaib AH, Ganzella M, Giannone G, et al. Synapsin condensation controls synaptic vesicle sequestering and dynamics Abstract Nature Communications. 2023;14(1). 10.1038/s41467-023-42372-6. 10.1038/s41467-023-42372-6 PMC10593750 37872159

[28] Takamori S, Holt M, Stenius K, Lemke EA, Grønborg M, Riedel D, et al. Molecular anatomy of a trafficking organelle. Cell. 2006;127:831–46. 17110340 10.1016/j.cell.2006.10.030

[29] Muzzopappa F, Hummert J, Anfossi M, Tashev SA, Herten D-P, Erdel F. Detecting and quantifying liquid–liquid phase separation in living cells by model-free calibrated half-bleaching. Nat Commun. 2022;13:7787. 36526633 10.1038/s41467-022-35430-y PMC9758202

[30] Imasaki T, Kikkawa S, Niwa S, Saijo-Hamano Y, Shigematsu H, Aoyama K, et al. CAMSAP2 organizes a γ-tubulin-independent microtubule nucleation centre through phase separation. Elife. 2022;11:e77365. 35762204 10.7554/eLife.77365 PMC9239687

[31] Song X, Yang F, Yang T, Wang Y, Ding M, Li L, et al. Phase separation of EB1 guides microtubule plus-end dynamics. Nat Cell Biol. 2023;25:79–91. 36536176 10.1038/s41556-022-01033-4

[32] Duan D, Lyu W, Chai P, Ma S, Wu K, Wu C, et al. Abl2 repairs microtubules and phase separates with tubulin to promote microtubule nucleation. Curr Biol. 2023;33:4582-4598.e10. 37858340 10.1016/j.cub.2023.09.018 PMC10877310

[33] Nagy A, Baker RR, Morris SJ, Whittaker VP. The preparation and characterization of synaptic vesicles of high purity. Brain Res. 1976;109:285–309. 132227 10.1016/0006-8993(76)90531-x

[34] Huttner WB, Schiebler W, Greengard P, De Camilli P. Synapsin I (protein I), a nerve terminal-specific phosphoprotein. III. Its association with synaptic vesicles studied in a highly purified synaptic vesicle preparation. J Cell Biol. 1983;96:1374–88. 6404912 10.1083/jcb.96.5.1374 PMC2112660

[35] Ahmed S, Holt M, Riedel D, Jahn R. Small-scale isolation of synaptic vesicles from mammalian brain. Nat Protoc. 2013;8:998–1009. 23619891 10.1038/nprot.2013.053

[36] Hoffmann C, Sansevrino R, Morabito G, Logan C, Vabulas RM, Ulusoy A, et al. Synapsin condensates recruit alpha-synuclein. J Mol Biol. 2021;433:166961. 33774037 10.1016/j.jmb.2021.166961

[37] Rizzoli SO, Bethani I, Zwilling D, Wenzel D, Siddiqui TJ, Brandhorst D, et al. Evidence for early endosome-like fusion of recently endocytosed synaptic vesicles. Traffic. 2006;7:1163–76. 17004320 10.1111/j.1600-0854.2006.00466.x

[38] Schmidt CC, Vasic V, Stein A. Doa10 is a membrane protein retrotranslocase in ER-associated protein degradation. Elife. 2020;9:e56945. 32588820 10.7554/eLife.56945 PMC7319771

