# Supplementary material for: Brain-derived synaptic vesicles have an intrinsic ability to sequester tubulin
Source: BMC Biol. 2025 Nov 14;23:340. doi: 10.1186/s12915-025-02464-9 (PMC12619239; doi:10.1186/s12915-025-02464-9)
Supplement: Supplementary file 1 — Additional file 1: Fig. S1. Tubulin accumulates in SV-enriched areas. Fig. S2. Pearson correlation coefficients of tubulin and various organelles’ intensity. Fig. S3. Comparison of average line profiles of tubulin and SV marker intensities of TRRs detected in samples using different labeling approaches [file 12915_2025_2464_MOESM1_ESM.pdf]

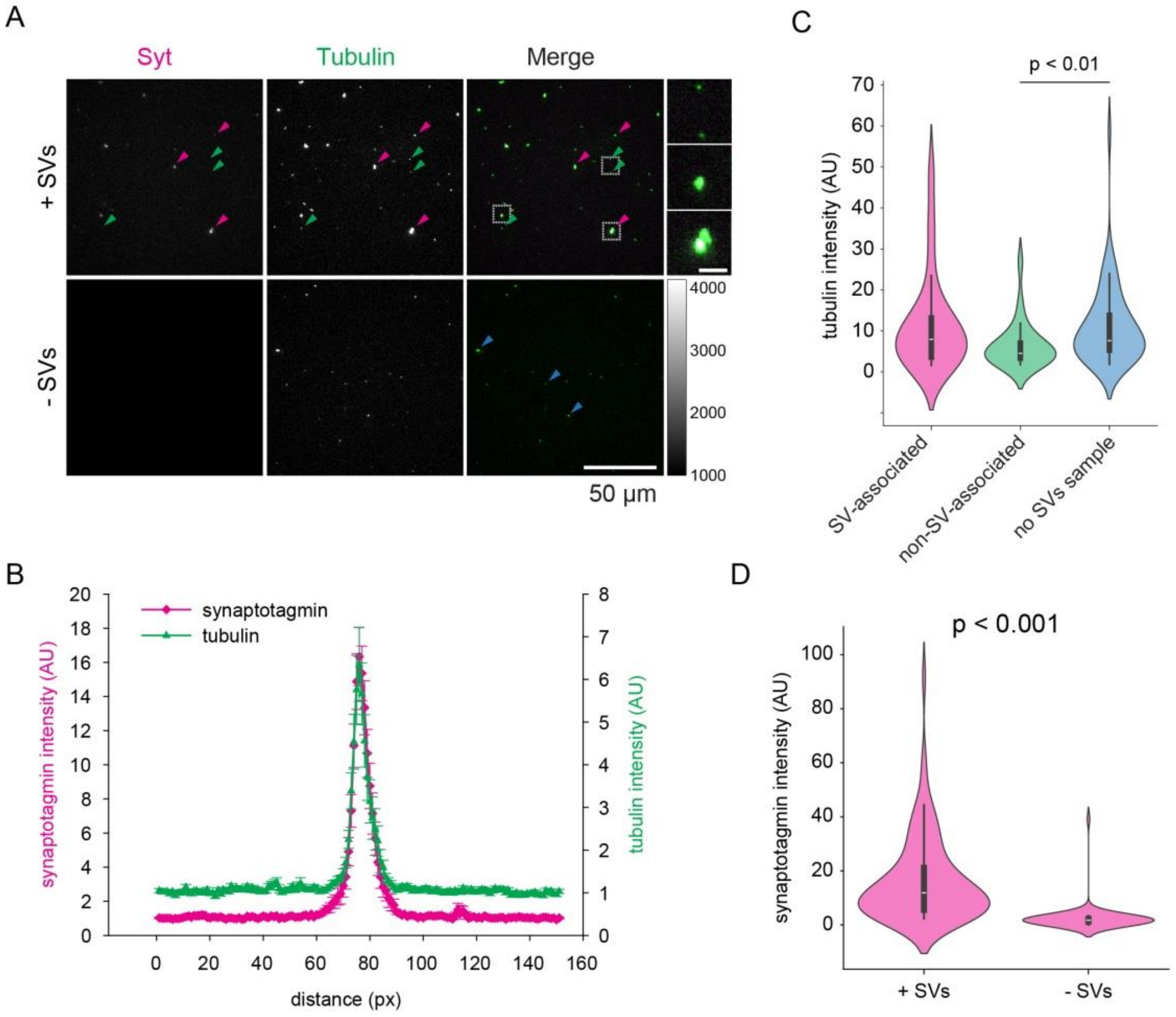

Figure S1. Tubulin accumulates in SV-enriched areas. A. Widefield view of tubulin distribution in the presence and in the absence of synaptic vesicles. Colored arrowheads indicate examples of different tubulin-rich regions (TRRs), which are analysed in panel C. Inset scale bar: 5 µm**. B.** Average line scans of tubulin signals overlapping with SVs. **C.** Comparison of tubulin intensity in different TRRs, based on their association with the SVs, as determined by the presence of the anti-synaptotagmin signal. Refer to panel A for examples: magenta arrowheads indicate TRRs associated with SVs, green arrowheads – TRRs not associated with SVs in a sample containing SVs, and blue – TRRs observed in the samples without SVs. Data from 3 independent experiments, 48 SV-associated TRRs, 27 SV-non-associated, and 60 TRRs in a sample lacking SVs were quantified. Indicated significance bracket: Kruskal-Wallis test, $p = 0.0087$. **D.** Synaptotagmin intensity in TRRs in samples with or without SVs. Data from 3 independent experiments, 86 regions from samples containing SVs and 60 regions from samples not containing SVs were quantified. Wilcoxon rank sum test, $p = 4.888\times10^{-21}$.

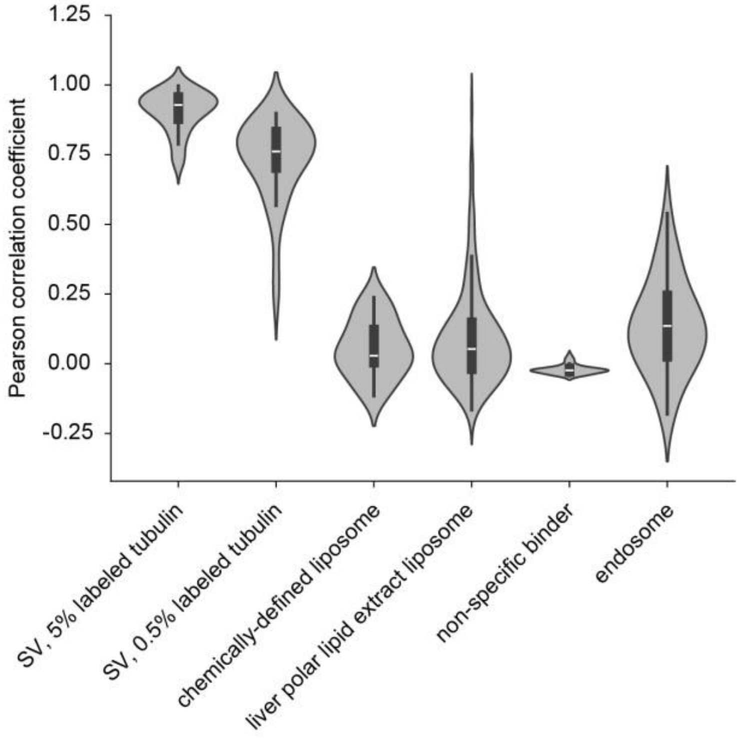

Figure S2. Pearson correlation coefficients of tubulin and various organelles' intensity. Each data point represents the Pearson R value, calculated for the two linescans in the tubulin and organelle images.

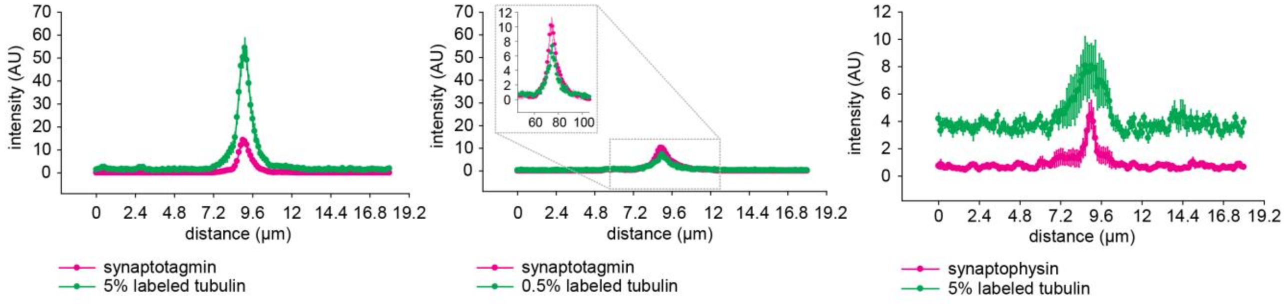

Figure S3. Comparison of average line profiles of tubulin and SV marker intensities of TRRs detected in samples using different labeling approaches. Decreasing the fraction of fluorescently labeled tubulin or targeting another SV protein does not affect the relative distribution of SVs and tubulin.
